# Assessment of Ovarian Cancer Tumors Treated with Intraperitoneal Cisplatin Therapy by Nanoscopic X-ray Fluorescence Imaging

**DOI:** 10.1038/srep29999

**Published:** 2016-07-21

**Authors:** Brecht Laforce, Charlotte Carlier, Bart Vekemans, Julie Villanova, Rémi Tucoulou, Wim Ceelen, Laszlo Vincze

**Affiliations:** 1 X-ray Microspectroscopy and Imaging Group (XMI), Ghent University, Krijgslaan 281 S12, B-9000 Ghent, Belgium; 2 Department of Surgery, Laboratory of Experimental Surgery, Ghent University Hospital, Ghent B-9000, Belgium; 3 European Synchrotron Radiation Facility (ESRF), FR-38043 Grenoble Cedex, France

## Abstract

Ovarian cancer is amongst the most common types of cancer in women, with a relatively low overall cure rate of approximately 30%. This is therefore an important incentive to urge for further research in order to maximize the chances of survival for these patients. Intraperitoneal chemotherapy with Cisplatin is an effective treatement for ovarian cancer; however, many questions still remain concerning the ideal treatment protocol and tumor resistance towards the drug, which should be resolved for optimal application of this therapy. For the first time *in-vivo* grown tumors treated with both hyper- and normothermic intraperitoneal chemotherapy have been studied using nano-XRF spectroscopy to examine the platinum (Pt) distribution within the analyzed tissues. These measurements prove Pt resides predominantly outsides the cancer cells in the stroma of the tissue. These findings indicate the resistance mechanism of the cancer cells prevents Cisplatin from diffusing through their cell membranes. This is an important addition to the existing knowledge on the resistance mechanism providing insights which might help to overcome this effect. In our aim to find the optimal treatment protocol, no significant differences were found between the two examined procedures. A more extensive data set will be needed to draw definite conclusions.

Worldwide, ovarian cancer is one of the most common types of cancer in women, according to a study published by the International Agency for Research on Cancer in 2008^1^. Currently, the overall cure rate for patients with ovarian cancer is approximately 30%^2^. Intraperitoneal (IP) chemotherapy with cisplatin has been shown to be an active treatment for stage III disease^3^,^4^,^5^. However, no standardized protocol for this treatment exists today^6^. Furthermore, the tumor cells develop resistance towards the drug relatively quickly^7^,^8^,^9^,^10^,^11^,^12^. By studying the sub-cellular distribution of Pt in tumor tissue after IP cisplatin chemotherapy, we aim to shed light on the underlying elemental correlations in the treated tumor tissue.

Previous studies investigating the distribution of cisplatin in tumors using elemental imaging techniques revealed the overall dispersion of Pt in the tissue and yielded crucial information on the penetration of the cisplatin drug^13^,^14^,^15^,^16^,^17^. However, these experiments lacked the necessary spatial resolution to investigate the platinum (Pt) distribution in the tumor cells in detail and currently no data based on *in vivo* models are available.

X-ray fluorescence (XRF) spectroscopy is a powerful technique for elemental analysis used in a wide range of scientific disciplines. Its non-destructive character makes it especially attractive when a broad spectrum of analytical methodologies is to be applied on the same sample, in order to gain a maximized amount of information. Synchrotron radiation based nano-XRF analysis yields high resolution data with a very high elemental sensitivity. Recently, several institutes have developed XRF nano-probes where elemental imaging with resolution levels reaching a few tens of nanometer is achievable^18^,^19^,^20^,^21^,^22^. One of the latest additions to this field of nano-imaging synchrotron radiation facilities is the ID16 beamline at the European Synchrotron Radiation Facility (ESRF) in Grenoble, France, which was commissioned in the period 2014–2015 and incorporates two nano-probe end stations: the ID16A Nano Imaging (NI) and ID16B Nano Analysis (NA) lines^21^,^23^.

This study aimed at obtaining information on the cisplatin-tissue interaction on the sub-cellular level based on *in vivo* models using quantitative XRF analysis. Athymic nude mice were injected bilaterally with SKOV3-LUC IP1 ovarian cancer cells. Two weeks after the injection, tumors of approximately 3–5 mm in diameter had grown. Subsequently, the mice were treated with IP cisplatin (0.7 mg/mouse) at 2 different temperatures. The ID16B beamline of the ESRF was used to investigate the distribution of Pt atoms, as indicative element for the presence of the cisplatin drug, in the tumor nodules excised from the mice. The nanoscopic imaging capabilities at the ID16B beamline of the ESRF enabled us to visualize the exact locations of Pt accumulation inside the tumor tissue on the sub-cellular spatial resolution level. This information is crucial when studying the efficiency of the drug, since only Pt interacting with the DNA of the tumor cells, present in the nucleus, is able induce apoptotic cell death^12^,^24^.

## Results

### Nanoscopic imaging of tumor cells

**Figure 1:**
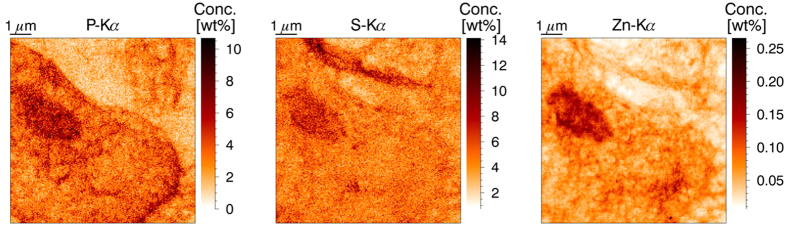
XRF elemental maps of P, S and Zn. Scanned area 9 × 9 μm, beam dimensions 50 × 50 nm^2^, 50 nm step size, measurement time 0.1 s per point, primary beam energy 17.5 keV. A tumor cell (nucleus and part of the cell membrane) can be discerned in the lower left part of the image.

Using the state-of-the-art ESRF ID16B XRF nano-probe, combined with a fast scanning procedure, the distribution of a wide range of major and trace elements present in the tumor nodules was imaged in a non-destructive manner. Figure 1 represents the elemental maps of P, S and Zn of a 9 by 9 μm area of tumor tissue. A part of a large tumor cell is present in the lower left part of the image. The membrane structures of cells contain phospholipids, causing the cell membrane and nucleus to be well-defined in the P-image. Sulfur is more uniformly distributed throughout the tissue and is present due to its role in protein bridges. The stroma of the tumor tissue proves to be relatively rich in S compared to e.g. P and Zn and shows elongated, thin areas with heightened sulfur content. Zinc is an important trace element for several biological functions (e.g. as cofactor in enzymatic processes or DNA recognition and binding). As such it is present mainly in the nucleus, distributed in a heterogeneous way, with the euchromatine being less densely packed than the heterochromatine and thus showing a lower elemental concentration in the corresponding XRF maps.

**Figure 2:**
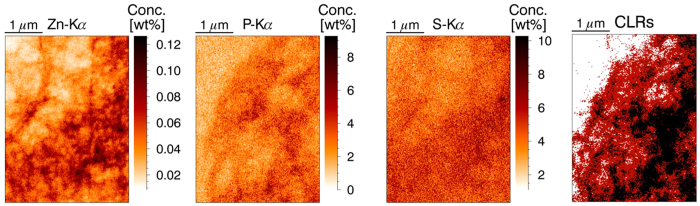
High resolution XRF elemental maps of P, S and Zn. Scanned area 4.5 × 3.2 μm, beam dimensions 50 × 50 nm^2^, 20 nm step size, measurement time 0.1 s per point. CLRs image shows the result of k-means clustering into three clusters (white = cytoplasm, red = euchromatine, black = heterochromatine), primary beam energy 17.5 keV.

A zoom-in image on a part of the nucleus of a tumor cell with an even higher resolution (20 nm step size) is given in Fig. 2. The outline of the nucleus is apparent in the P-image, while sulfur is uniformly distributed. The Zn map shows a large amount of small structures inside the nucleus. These structures are the nucleoli, where the ribosome synthesis takes place. The last frame of Fig. 2 shows the result of k-means clustering on this data set^25^,^26^. A clear distinction can be made between the region outside the nucleus (the cytoplasm, white) the main matrix of the nucleus (red) and the nucleoli (black).

The elemental signature of the different structures of the tumor tissue enables us to classify the other elements based on their correlation with P, S and Zn. A strong correlation with phosphorous is indicative for elements present in the tumor cells. Furthermore, in case a correlation with zinc is apparent, the element can be linked to the nucleus. Low correlation with phosphorous, but a connection with sulfur indicates an element mainly present in the stroma of the tissue. This information will be used to assay the predominant location of cisplatin in the tumor sections.

### Correlation between Pt and other (trace) elements

**Figure 3:**
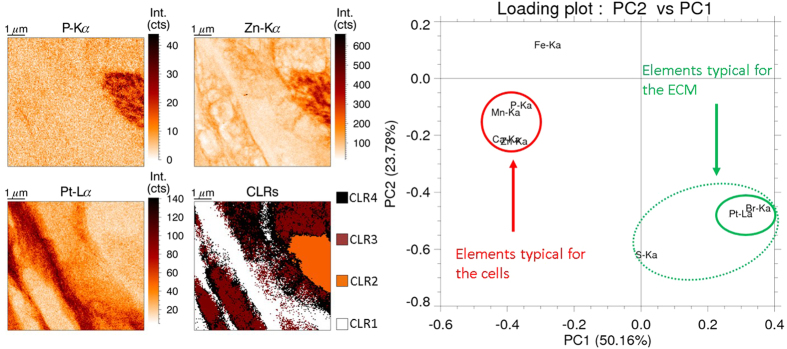
Data mining results from XRF maps. Scanned area 16 × 16 μm, beam dimensions 50 × 50 nm^2^, 50 nm step size, measurement time 0.1 s per point, primary beam energy 17.5 keV. K-means clustering (left): combining the P, Zn and Pt elemental maps with the cluster map shows Pt (CLR1, white) resides outside the nucleus (CLR2, orange) and cytoplasm (CLR3, brown). PCA (right): strong link between Pt and Br and P, Mn, Ca and Zn.

Principal component analysis (PCA) and k-means clustering were applied to the analyzed XRF maps, looking for links and correlations between the detected elemental constituents. Fig. 3 shows the results of k-means clustering and PCA analysis on a tumor section treated at 37 °C with IP cisplatin. The clustering shows Pt resides preferentially outside the tumor cells, in the stroma of the tissue. Quantification showed the Pt concentration outside the cells is twice as large as the Pt content of the cancer cells. This is confirmed by the PCA analysis, where Pt shows a strong link with Br and to a lesser extent with S (indicated in green), while no apparent correlation exists between Pt and P or Zn, the elements typical for the cytoplasm and the nucleus of the cell. Iron, Manganese and calcium were also investigated with the above data mining procedures. The latter two elements show a strong correlation with P and Zn (red circle in Fig. 3), indicating a predominant presence in the cells, while Fe has no correlation with any of the other elements, although it is among the essential elements typical for the cells, unlike the Pt-Br group.

There are several hypotheses as to why Pt resides predominantly in the stroma. First of all, the tumors were excised and fixated immediately after the 1 h IP treatment. Potentially this time frame is too short for the cisplatin drugs to enter the cancer cells, causing it to be present mainly in the looser connective tissue in between the cells. Secondly, the cancer cells were injected into the mice in a matrigel solution. It is possible that the affinity of cisplatin to the scaffold is too high, causing it to adsorb to components of the matrigel instead of actively entering the cancer cells. Thirdly, the fixation process using formaldehyde removes any unbound Pt. This was a conscious choice, enabling us to investigate only the active cisplatin molecules by washing away the unbound residues. However, the time scale of 1 h might be too short for the cisplatin entering the cancer cells to adhere to internal structures, thus causing it to be removed during sample preparation. Lastly, it is possible these observations are part of the resistance mechanism of the cancer cells which is effectively stopping the cisplatin from diffusing through the cell membrane thus keeping it in the stroma of the tissue. Further research has to be performed to clarify the reason behind the predominant presence of Pt in the stroma. These experiments will need to investigate the role of the matrigel matrix and the time scale of the different processes.

The second observation, the strong link between Pt and Br derived via PCA, can be explained by the chemical properties of the latter element. In the cisplatin molecule, a central Pt atom is linked to two NH3 functions and two Cl atoms. Both being halogens, Br and Cl have comparable chemical properties and thus show comparable affinity towards the Pt atom of the cisplatin molecule, causing bromine, being a known trace element in animal tissue, to concentrate around the same positions as the anti-cancer drug.

### Effect of treatment protocol on the Pt distribution

**Figure 4:**
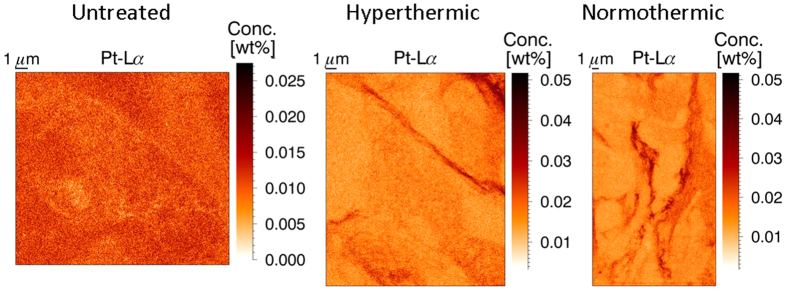
XRF Pt elemental maps of an untreated (left), hyperthermic (middle) and normothermic (right) treated tumor sample. The two treated samples yield very similar XRF maps, with Pt present in string-like structures, while the untreated sample shows a quite uniform Pt background signal.

All XRF scans were performed at the same distance of the peritoneal rim of the tumor samples (75 μm), facilitating quantitative comparison of the different results. There were three types of samples to be evaluated: untreated tumor tissue, tissue treated with hyperthermic (41 °C) IP cisplatin, and tissue treated with IP cisplatin at 37 °C (normothermic). Figure 4 shows an XRF map for each of these categories, while the table in Table 1 gives the average data for all measured samples (2 untreated, 5 hyperthermic and 7 normothermic treated). In the untreated sample, a relatively low amount of Pt is expected when compared to the treated samples. The fitting of a very small peak (below the detection limits) leads to numerical results with a very high standard deviation. Hence the values for Pt of the untreated samples were not incorporated into Table 1. The hyper- and normothermic treated samples show very similar results, both visually and numerically. It is obvious from Fig. 4 the Pt barely entered the tumor cells, but is mainly present in string-like structures in the stroma of the tissue for both treatment protocols. No statistical significant difference in Pt concentrations could be found for both treatments at this penetration depth. The concentration of the other elements of interest varies to a larger extent than for Pt. Further experiments should be conducted to draw any definite conclusions on the difference between the hyper- and normothermic intraperitoneal chemotherapy treatments, focusing on the penetration depth of the cisplatin drug in the tissue and possible differences in diffusion behavior on a larger timescale.

Overall, the results of this study demonstrate the capabilities of synchrotron radiation based nano-XRF spectroscopy to examine the elemental composition and distribution of this type of biomedical samples. The remaining questions urge for further investigations, combining this technique with the wide spectrum of other X-ray based nano-analysis techniques available at synchrotron facilities (e.g. nano-XAS) to study Pt resistance mechanisms of cancer cells and the different treatment protocols.

## Methods

### Tumor model and IPC treatment

The Animal Ethics Committee of the Faculty of Medicine at Ghent University (ECD 15/51) approved the animal experiments. The animal experiments were performed according to the Belgian and European legislature on animal welfare.

Ten athymic, nude-foxn1nu female mice (age 6 weeks) with an avarage weight of 20 g were conditioned one week before the start of each study. The mice were injected bilaterally, subperitoneally with 5.0 × 10^5^ SKOV3-LUC IP1 ovarian cancer cells, dissolved in 50 μl of matrigel^27^. Two weeks after the injections, when the two tumor nodules in each mouse were approximately 3–5 mm in diameter, mice were treated with IP cisplatin (0.7 mg/mouse) using a closed perfusion circuit during 60 minutes. Five mice were treated at an IP temperature of 37–38 °C (normothermic group), and five other mice were treated at 41–42 °C (hyperthermic group). After the IP treatment, mice were sacrificed and the tumor nodules were excised, fixed in 4% paraformaldehyde overnight and embedded in paraffin (Klinipath). Three consecutive paraffin slices with a thickness of 2 μm were cut with a HM 355S microtome (Thermo Fisher scientific, Ghent, Belgium) The first and third slice were Hematoxylin and eosin (H&E) stained, while the second paraffin slice was positioned on ultralene^®^ foil (SPEX SamplePrep, Metuchen, USA) for XRF-analysis. These samples were mounted on polyether ether ketone (PEEK) slides, with a central opening.

### XRF imaging at ID16B (ESRF, Grenoble, France)

The ESRF ID16B-NA beamline^23^ was used to image the elemental distributions in the tumor tissue sections. The long distance between the experimental station and the X-ray source at this beamline, combined with a high demagnification Kirkpatrick-Baez (KB) mirror system, yields a spot size of 50 by 50 nm. The energy of the incident X-ray photons is 17.5 keV (±1%) with a photon flux of approximately 2 × 10^11^ ph/s.

**Figure 5:**
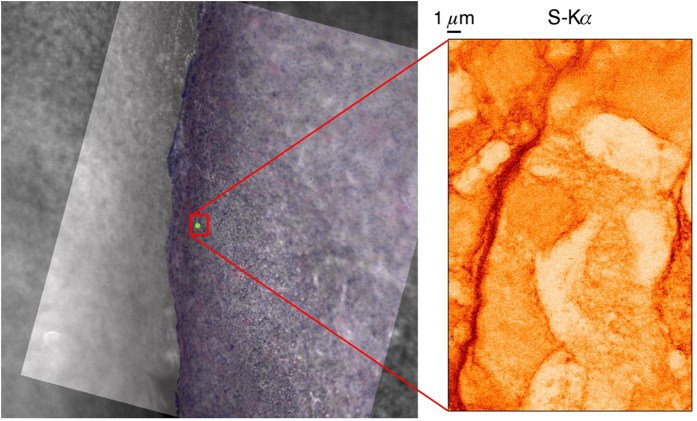
Sample positioning procedure: H&E stained microscope image is rotated and aligned with online microscope view to select the ROI (left) yielding direct correlation with XRF mappings (e.g. S on the right). Scanned area 18 (H) × 28 (V) μm, beam dimensions.

A pierced mirror microscope is used to monitor the sample positioning and to define the scan areas. The tumor tissue was not stained to avoid elemental contamination; however, this resulted in a poor contrast of the cells using the optical beamline microscope, requiring the development of a dedicated sample positioning procedure. In order to retrieve the selected ROIs, three adjacent sections were made. The outer two were H&E stained and imaged with an optical microscope. These images were used to create an overlay for the beamline microscope imaging software. By rotating and aligning the overlay image with the view from the beamline microscope, accurate positioning of the sample was possible relatively fast and easy, as demonstrated in Fig. 5.

The incident X-ray beam is oriented perpendicularly to the scanning plane defined by the sample positioning system, requiring the two 3-element SDD detectors to be positioned slightly off the ideal angle of 90° to the primary beam (13° deviation). The effect of this offset is limited, since the centers of the multi-element detectors are still placed in the plane of polarization; the outer detectors have a deviation of 26°.

Due to physical limitations of the XRF methodology, only elements with an atomic number larger than 12 (i.e. starting from aluminum) can be detected. To determine the sensitivity of the instrument towards the detectable elements, several mappings were performed on NIST SRM 1577b and 1577c reference materials (100 μm thickness) which were then employed to determine the elemental detection limits (DLs). The detection limit of an element is defined by the concentration value at which the detected peak intensity can be statistically distinguished from the random fluctuations of the corresponding background at a confidence level of 3 s.d. The resulting detection limit (DL) formula is as follows:


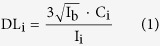


**Figure 6:**
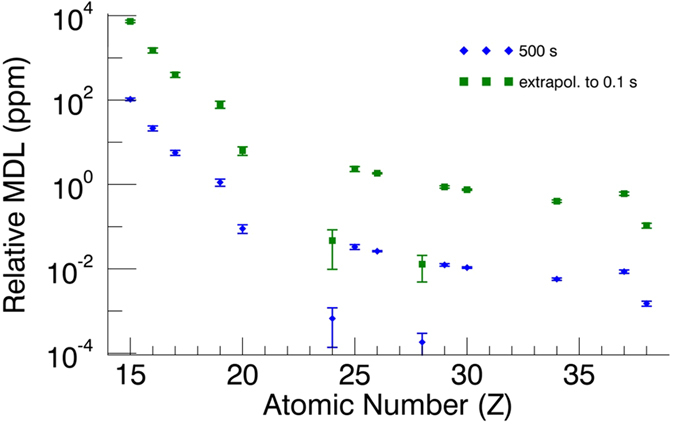
Rel. MDLs of the ID16B nano-probe for NIST SRM 1577c standard material, 500 s and 0.1 s measurement time.

The value *DL*_*i*_ is expressed for each element *i* in units of concentration, *I*_*b*_ is the intensity of the background signal, *C*_*i*_ is the certified concentration of element *i* in the NIST SRM and *I*_*i*_ is the measured peak intensity of the element in the standard reference material. The results of the DL calculations were extrapolated towards 0.1 s, the dwell time used during the scanning XRF experiments, yielding a detection limit of 0.73 wt% for P, 0.15 wt% for S, 6.28 ppm (i.e. μg/g) for Ca and 0.75 ppm for Zn (Fig. 6). The above described procedure for determining the detection limit could not be applied for Pt, since no suitable XRF reference material containing this element was available.

For Pt, the detection limit was calculated via a Monte Carlo simulation aided quantification procedure^28^ and was found to be 0.41 ppm. Using the relevant properties of the sample (density and thickness) and the incident beam (footprint on the sample), this corresponds to an absolute detection limit of 2.20 fg Pt within the illuminated volume in the sample. To verify if the simulation yielded reliable results for the detection limit, the DLs of Ca and Zn were also calculated. A detection limit of 3.91 ppm for Ca and 0.44 ppm for Zn were obtained, which is in a relatively good agreement with the experimental DLs.

Large areas were scanned with 50 nm step size, while detail images were taken with steps of 20 nm. The dwell time was optimized to achieve an optimal tradeoff between short measurement time and adequate statistics and was put at 0.1 s/point.

### XRF data analysis and quantification

The XRF spectra were fitted using the iterative least squares algorithm AXIL^29^,^30^. In-house developed data analysis software was employed to process the fitted spectra, creating elemental image files and performing the k-means clustering and PCA data mining procedures^26^.

**Figure 7:**
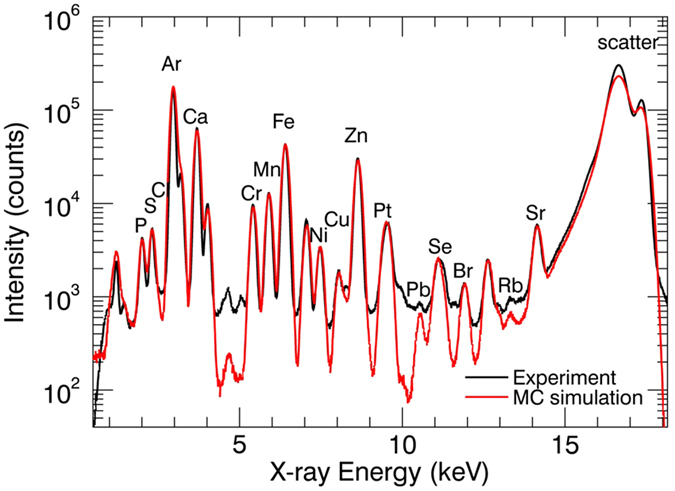
Monte Carlo (MC) simulation aided quantification procedure: the experimental spectrum (black) coincides with the simulated spectrum (red). Using the response parameters of the MC simulation, intensity can be linked to concentration for all measurements.

The XRF data were quantified based on a Monte Carlo (MC) simulation aided procedure^28^,^31^,^32^,^33^. Measurements of a NIST SRM 1577c bovine liver standard were used to determine the exact characteristics and calibration parameters of the ESRF ID16B XRF spectrometer. Next, the sum spectrum of an XRF mapping of a tumor section was used to establish the (average) spectral response characteristics of the samples. An example in Fig. 7 represents the experimental and the corresponding simulated XRF spectra. The simulation follows the experiment closely, with the only deviation being a slight underestimation of the background and the scatter peaks. The underestimation of the background most likely results from the insufficiently known detector response function, while the difference in scatter intensities may originate from uncertainties in the estimated degree of linear polarization of the focused beam. It has been verified using reference materials that the quantification results are not influenced significantly by these two differing spectral regions. Based on the simulated spectra, therefore, the relation between intensities and concentrations could be obtained with satisfactory accuracy and used to quantify the XRF scans.

## Additional Information

**How to cite this article**: Laforce, B. *et al.* Assessment of Ovarian Cancer Tumors Treated with Intraperitoneal Cisplatin Therapy by Nanoscopic X-ray Fluorescence Imaging. *Sci. Rep.* **6**, 29999; doi: 10.1038/srep29999 (2016).

## Figures and Tables

**Table 1:** Average elemental concentration in the tumor samples as quantified with MC-aided XRF analysis.

	P (wt%)	S (wt%)	Zn (wt%)	Pt (wt%)
Untreated	2.46 ± 1.04	5.03 ± 0.73	0.025 ± 0.022	<DL
Hyperthermic	2.91 ± 1.08	6.48 ± 1.25	0.045 ± 0.015	0.016 ± 0.002
Normothermic	2.21 ± 0.61	4.68 ± 1.10	0.036 ± 0.014	0.018 ± 0.007

The data set comprised 2 untreated, 5 hyperthermic and 7 normothemic treated tumor samples, examined at 75 μm from the peritoneal membrane.
